# Threshold-Dependent BMP-Mediated Repression: A Model for a Conserved Mechanism That Patterns the Neuroectoderm

**DOI:** 10.1371/journal.pbio.0040313

**Published:** 2006-09-12

**Authors:** Claudia Mieko Mizutani, Néva Meyer, Henk Roelink, Ethan Bier

**Affiliations:** 1 Section of Cell and Developmental Biology, University of California San Diego, La Jolla, California, United States of America; 2 Department of Biological Structure, University of Washington, Seattle, Washington, United States of America; Stanford University, United States of America

## Abstract

Subdivision of the neuroectoderm into three rows of cells along the dorsal-ventral axis by neural identity genes is a highly conserved developmental process. While neural identity genes are expressed in remarkably similar patterns in vertebrates and invertebrates, previous work suggests that these patterns may be regulated by distinct upstream genetic pathways. Here we ask whether a potential conserved source of positional information provided by the BMP signaling contributes to patterning the neuroectoderm. We have addressed this question in two ways: First, we asked whether BMPs can act as bona fide morphogens to pattern the *Drosophila* neuroectoderm in a dose-dependent fashion, and second, we examined whether BMPs might act in a similar fashion in patterning the vertebrate neuroectoderm. In this study, we show that graded BMP signaling participates in organizing the neural axis in *Drosophila* by repressing expression of neural identity genes in a threshold-dependent fashion. We also provide evidence for a similar organizing activity of BMP signaling in chick neural plate explants, which may operate by the same double negative mechanism that acts earlier during neural induction. We propose that BMPs played an ancestral role in patterning the metazoan neuroectoderm by threshold-dependent repression of neural identity genes.

## Abstract 2

This study provides evidence that graded bone morphogenic proteins (BMPs) act as morphogens in neuroectoderm patterning in*Drosophila* and chick by repressing expression of neural identity genes in a threshold-dependent fashion.

## Introduction

Morphogen gradients play a central role in creating pattern during embryonic development [1,2]. Bone morphogenetic proteins (BMPs) are one of the best studied examples of morphogens and function in a conserved fashion to subdivide the early embryonic ectoderm into neural versus non-neural regions [3]. Following this role in establishing the primary ectodermal domains, BMPs and their antagonists, such as Short gastrulation (Sog)/Chordin (Chd), interact in a graded fashion to establish a series of nested gene expression domains in the non-neural ectoderm. While this BMP-mediated partitioning of the non-neural ectoderm has been analyzed in quantitative detail in *Drosophila* [4–6], relatively less is known about how patterning is initiated within the neuroectoderm.

**Figure 1:**
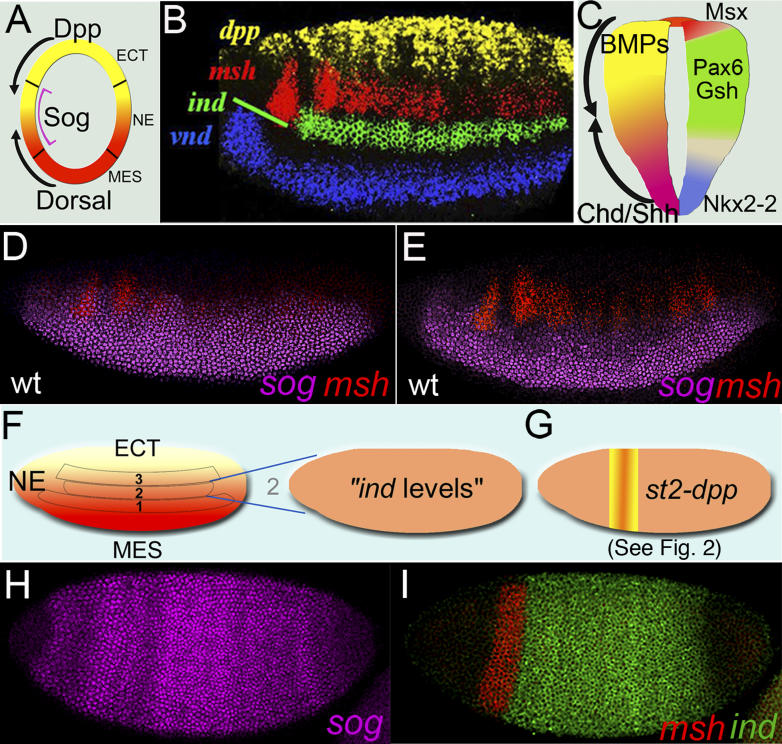
A Conserved Pattern of Gene Expression in the Neuroectoderm (A) Diagram indicating the relative positions of opposing BMP and Dorsal gradients in a transverse cross-section of a blastoderm stage *Drosophila* embryo. (B) Multiplex in situ staining of a *Drosophila* blastoderm stage embryo showing expression of *vnd, ind, msh,* and *dpp* along the DV axis. Dorsal is to the top and anterior to the left in this and subsequent figures. (C) Scheme indicating the relative expression domains of Nkx2.2, Gsh, Pax6, Msx1/2 as well as the BMP and Shh protein gradients in the vertebrate neural tube. (D and E) Dynamics of *sog* expression (purple) and *msh* expression (red). (D) In mid-blastoderm stage embryos, *sog* expression begins to fade from most dorsal cells of the neuroectoderm at the same time that *msh* expression is initiated as a partial stripe. (E) In slightly later embryos, the domains of *sog* and *msh* expression become nearly complementary. (F) Scheme for generating lateralized embryos with a uniform level of Dorsal adjusted to the level present in the mid-neuroectoderm (e.g. *ind*-expressing cells). These embryos were collected from females of the genotype *gd* ^7^ *sog* ^U2^/*gd* ^7^ *; dl* ^1^/+*; Tl* ^3^/+. (G) The same females were crossed to males carrying a homozygous insertion of an *st2-dpp* construct [75] to generate lateralized embryos expressing *dpp* in a stripe (see Figure 2). (H and I) Expression of neuroectodermal genes in lateralized embryos. (H) *sog* (purple). (I) *ind* (green) and *msh* (red). Note that the ring of *msh* expression directly abuts the domain of *ind* with no overlap and extends anteriorly beyond the domain of *ind* expression to approximately the same extent as observed in wild-type embryos (see [B]).

The neuroectoderm in *Drosophila* and vertebrate embryos is similarly subdivided into three conserved dorsal-ventral (DV) domains expressing the homeobox genes *ventral nervous system defective (vnd)*/*Nkx2.2*, *intermediate nervous system defective (ind)*/*Gsh*, and *muscle specific homeobox (msh)/Drop*/*Msx1/2* (Figure 1). These neural identity genes are expressed in ventral, intermediate, and dorsal domains (Figure 1A–1C) [3,7–10], respectively, and are required to specify neural fates [11–17]. In *Drosophila*, both loss-of-function and mis-expression experiments have revealed that neural-identity genes cross-regulate each other in a ventral-dominant fashion wherein ventrally expressed genes repress expression of more dorsal ones [9,12,16,18], a mechanism likely to have been conserved in vertebrates [19–21].

An important unresolved question is whether subdivision of the neuroectoderm is accomplished by a conserved process or has arisen as a consequence of convergent evolution [7]. As in the case of anterior-posterior (AP) patterning, where apparently species-specific upstream processes activate *HOX* genes in a conserved order along the AP axis [22], distinct pathways have been implicated in DV patterning of the neuroectoderm. The Dorsal gradient in *Drosophila* plays a central role in establishing the DV axis in *Drosophila* [23] and acts directly to initiate expression of *vnd* [24] and *ind* [25], while the Sonic Hedgehog (Shh) gradient in vertebrates patterns the ventral and lateral regions of the neural tube [19,23,26] (Figure 1A and 1C).

It has been suggested that BMP signaling might provide a conserved source of positional information along the DV axis in the neuroectoderm of both vertebrates and invertebrates. BMPs are expressed in the adjacent non-neural ectoderm, and following their early role during segregation of neural versus non-neural domains, they regulate expression of genes in the neuroectoderm. Disrupting the function or the extracellular distribution of BMPs or their antagonists such as Sog/Chd or Noggin leads to neuroectodermal patterning defects [27–31]. In addition, BMPs and Sog/Chd are expressed in the same relative orientation with regard to the domains of neural identity genes in the adjacent non-neural ectoderm (Figure 1A and 1C) [3,7,8,10,32].

Despite the similarities mentioned above, there are two apparent differences between vertebrates and invertebrates that argue against a common ancestral role for BMPs in patterning the neuroectoderm. First, it has been proposed that BMPs activate the expression of neural genes in the dorsal region of the vertebrate neural tube [33,34], whereas this pathway has only been reported to repress neural gene expression in *Drosophila* [18,27,35,36]. Furthermore, although BMPs appear to function as morphogens to pattern the dorsal region of the vertebrate neural tube, there is no evidence that BMPs act in a similar dose-dependent fashion in the *Drosophila* neuroectoderm. In fact, existing studies suggest that BMPs play little, if any, role in establishing the order of neural identity gene expression, but function rather to consolidate cell fates choices [8,18,27,37]. It is possible that these prior studies failed to demonstrate a coherent role of BMPs in organizing the neuroectoderm due to the predominant influence of Dorsal in establishing the DV axis. Thus, it remains an open question whether BMPs play conserved or convergently evolved roles in establishing neuroectodermal cell fates in vertebrates and invertebrates.

In the current study, we asked whether BMP signaling alone can provide positional information for subdividing the neuroectoderm. We created *Drosophila* embryos devoid of normal DV polarity and determined whether neuroectodermal patterning could be restored in response to an ectopic Decapentaplegic (Dpp) gradient provided along the AP axis. These experiments reveal that BMP signaling acts in a graded fashion to preferentially repress the expression of more ventral neural identity genes within the intermediate and dorsal regions of the neuroectoderm. We employed a similar experimental strategy to generate cells of uniform DV identity in chick neural tube explants grown in culture. In this case, the patterning effect of BMPs was assessed by adding increasing doses of BMPs to the media or by co-culturing BMP expressing cells with neural plate explants. As in *Drosophila* embryos, we observed that BMPs could act in a dose-dependent fashion to recreate DV patterning in the absence of other graded cues. Such graded BMP-mediated repression in combination with cross-regulatory repression among neural identity genes can provide information required to subdivide the neuroectoderm into discrete domains and may once have been sufficient to organize the entire ectodermal DV axis of metazoan ancestors.

## Results

The neural identity genes *vnd, ind,* and *msh* are expressed in a series of non-overlapping DV domains in the *Drosophila* embryo (Figure 1B). These genes are expressed in a highly dynamic fashion and are activated in a ventral-to-dorsal sequence [9,18]. The BMP antagonist Sog is expressed throughout the neuroectoderm [38] prior to the activation of neural identity gene expression and fades dorsally (Figure 1D and 1E) as the Dorsal gradient collapses [38,39]. By the time *msh* is expressed in a single contiguous dorsal stripe, *sog* expression is largely lost from these dorsal-most cells (Figure 1E). During this same period, the BMP2/4 homolog Dpp is expressed in adjacent dorsal cells (Figure 1B), where it represses the expression of neural genes and acts in a graded fashion to pattern the non-neural ectoderm. It is possible that Dpp also signals to the neuroectoderm, although previous single and double mutant analyses of the *dpp* pathway have not resolved whether Dpp acts in a graded fashion to help establish the order of the neural domains. In none of these studies, was it possible to sort out the contribution of BMP signaling from that of the Dorsal gradient (Figure 1A)*.* To answer whether Dpp acts as a morphogen to pattern the *Drosophila* neuroectoderm, we developed a system for selectively analyzing its effects in the absence of other DV cues.

### Separating the Effects of Graded BMP Signaling from that of the Dorsal Gradient

In order to separate the potential patterning effect of BMP signaling in *Drosophila* from that imposed by the Dorsal gradient, we designed a genetic system that allowed us to replace the normal ventral-to-dorsal gradient of nuclear Dorsal with a uniform neuroectodermal level of Dorsal along the entire DV axis of the embryo (Figure 1F). These lateralized embryos were created by first eliminating polarized DV maternal patterning acting upstream of Toll signaling and then adding back uniform adjusted levels of Dorsal across the entire DV axis using activated alleles of the Toll receptor. Uniform maternal Toll signaling was adjusted to specific levels using activated Toll alleles of differing strengths and by altering the dose of maternal Dorsal [40] (see Materials and Methods for details). In such lateralized embryos, we then tested the response of neural genes to an ectopic BMP gradient formed along the AP axis. This BMP gradient was created by expressing *dpp* under the control of the *even-skipped* stripe 2 enhancer of *dpp (st2-dpp)* construct (Figure 1G).

In lateralized embryos, pan-neuroectodermal markers such as *sog* are expressed around the entire circumference of the embryo (Figure 1H). As expected from the threshold-dependent activity of Dorsal [23], mesodermal, and dorsal ectodermal markers are absent in these same embryos (unpublished data). The consistent and uniform amounts of Dorsal produced in these lateralized embryos correspond to mid-neuroectodermal levels as revealed by expression of *ind* (Figure 1I) along the full DV axis and the absence of *vnd* expression (unpublished data). The AP limits of *ind* expression (Figure 1I) are similar to those in wild-type embryos (Figure 1B). Within this domain, *msh* expression is not detectable (Figure 1I), presumably because Ind is acting in a ventral-dominant fashion to repress it [9,16]. However, in more anterior cells abutting the *ind* domain, where *msh* expression normally extends further than *ind* (Figure 1B), *msh* is expressed in a ring around the embryo (Figure 1I). These initial studies indicate that both *ind* and *msh* can be expressed in mid-neuroectodermal lateralized embryos, and that Ind efficiently excludes *msh* from its domain.

### Dpp Represses *msh* and *ind* Expression in a Threshold-Dependent Fashion

**Figure 2:**
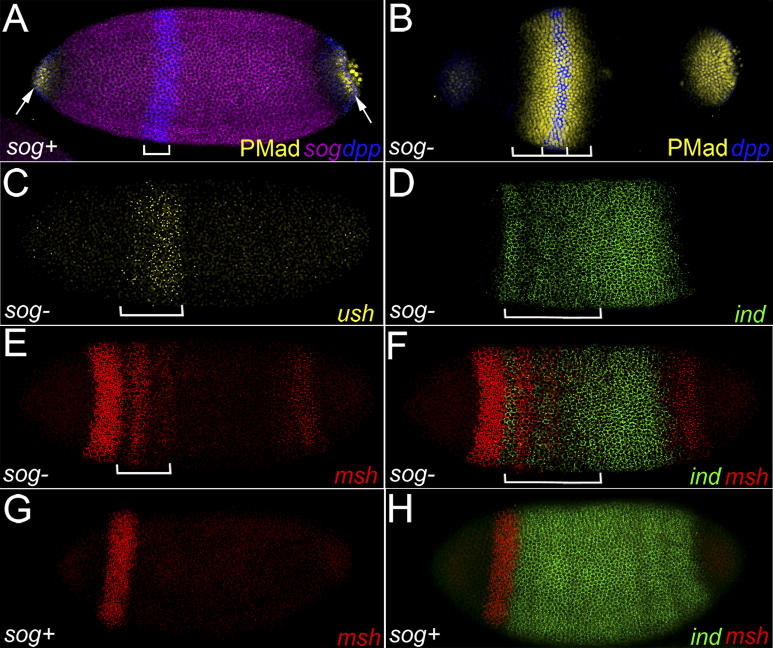
Threshold-Dependent Repression of *ind* and *msh* in Lateralized Embryos (A) *sog* (purple), *dpp* (blue), and pMAD (yellow) expression in a lateralized embryo carrying *st2-dpp*. Note that there is no activation of pMAD in the central stripe of *dpp* expression. pMAD activation at the poles (arrows) serves as a positive control for the staining. (B) *sog* (absence of purple), *dpp* (blue), and pMAD (yellow) expression in a *sog*− lateralized embryo carrying *st2-dpp*. The domain of pMAD activation (long bracket) extends considerably beyond the narrow stripe of *dpp* expression (short bracket). (C–F). Expression of *ush* (yellow), *ind* (green), and *msh* (red) in a *sog*−*; st2-dpp* lateralized embryo derived from *gd* ^7^ *sog* ^U2^/*gd* ^7^ *; dl* ^1^/+*; Tl* ^3^/+ mothers crossed to *yw; st2-dpp* males. (C) *ush*, which serves as a convenient marker for BMP activation, is induced in a domain (bracket) that is slightly broader than that of *st2-dpp*. (D) *ind* expression is repressed in a graded fashion along the AP axis extending approximately 20 cell diameters posterior to *st2-dpp* (bracket). (E) *msh* expression is activated in a pattern complementary to that of *ind* (bracket). This pattern exhibits modulation along the AP axis that is similar to the pattern of *msh* activation in wild-type embryos (see Figure 1D and 1E). (F) Merge of *ind* and *msh* expression patterns shown in (D and E) (bracket as in [D]). The restriction of *msh* expression to cells with very low levels of *ind* suggests that only modest levels of *ind* are necessary to repress *msh* expression. (G and H) Expression of *ind* (green), and *msh* (red) in a *sog*+*; st2-dpp* lateralized embryo. These embryos are siblings of the embryos described above in (C–F). *ush* expression is absent in this embryo. (G) *msh* expression is restricted to a narrow anterior stripe as it is in the absence of the *st2-dpp* element (compare with Figure 1H). (H) Merge of *msh* and *ind* expression showing that these gene expression domains are complementary (compare with Figure 1I).

Once we established conditions for reliably producing lateralized embryos, we tested whether it was possible to induce a graded Dpp response by crossing a *st2-dpp* construct into the lateralized background (Figure 2). The sole source of *dpp* expression in these embryos is provided by *st2-dpp*, except at the poles where endogenous *dpp* expression is independent of Dorsal regulation [41] (Figure 2A, arrows). The expected pattern of BMP pathway activation in such embryos, assessed by in situ phosphorylation of the signal transducer, phosphorylated form of Mothers against dpp (pMAD), is a broad band centered over the *st2*-*dpp* stripe [6]. We also assayed expression of the epidermal Dpp target gene *u-shaped (ush)* as a second marker for BMP activation. Because lateralized embryos ubiquitously express the BMP inhibitor *sog,* neither pMAD (Figure 2A) nor *ush* expression (unpublished data) could be detected near the stripe of *dpp* expression. However, when *sog* function was eliminated in *st2-dpp* lateralized embryos, pMAD was activated in a broad domain extending approximately eight cell diameters beyond the narrower *dpp* stripe (Figure 2B). In addition, *ush* expression was also activated in this region (Figure 2C). These results indicate that Dpp diffusing from a sharp stripe can elicit a graded response over significant distances.

We next examined the effect of graded Dpp activity on the relative patterns of *ind* and *msh* expression. We used multiplex in situ hybridization methods [42] to examine the simultaneous expression of *msh, ind,* and *ush,* while scoring for the *sog*+ versus *sog*− genotype of the embryos. These experiments revealed a clear dose-dependent repression of *ind* expression characterized by strong repression near the source of *dpp* and graded reduction in expression extending approximately 20 cell diameters posteriorly (Figure 2D, bracket). In contrast, the opposite effect was observed with regard to *msh* expression, resulting in its activation in cells expressing the lowest levels of *ind* (Figure 2E and 2F). In control *sog*+ lateralized embryos, where BMP signaling is blocked, *st2-dpp* had no discernable effect on the pattern or intensity of either *msh* or *ind* expression (Figure 2G and 2H; compare with Figure 1I). These results can be understood if Dpp signaling preferentially represses expression of *ind* in *sog*−; *st2-dpp* lateralized embryos, thereby relieving *ind*-mediated repression of *msh* in cells near the Dpp source. The induction of *msh* expression near the Dpp stripe followed by a zone of *ind* expression mimics the wild-type configuration of gene expression and provides the first evidence that BMP signaling can influence the pattern of neuroectodermal gene expression in the absence of other DV cues such as the Dorsal gradient. Similar long-range inhibition of *ind* and short-range induction of ectopic *msh* expression can be observed in *sog*−; *eve2-dpp* embryos with an intact Dorsal gradient (see Figure S1), indicating that *ind* is also likely to be more sensitive than *msh* to BMP-mediated repression in wild-type embryos (see also Figure S2). The fact that the zone of *ind* repression extends considerably further from the *dpp* stripe than the region of *msh* activation indicates that *msh* is not responsible for *ind* repression, consistent with existing evidence that *msh* does not regulate *ind* [9,16]. It seems likely, therefore, that BMP signaling acts directly to repress *ind* expression, as has been proposed previously [18]. These data support the prevailing ventral-dominant model for cross-regulation of neural identity genes, and exclude an alternative model in which Dpp signaling activates *msh,* which in turn inhibits *ind*.

### *vnd* Expression Is Also Sensitive to BMP-Mediated Repression

**Figure 3:**
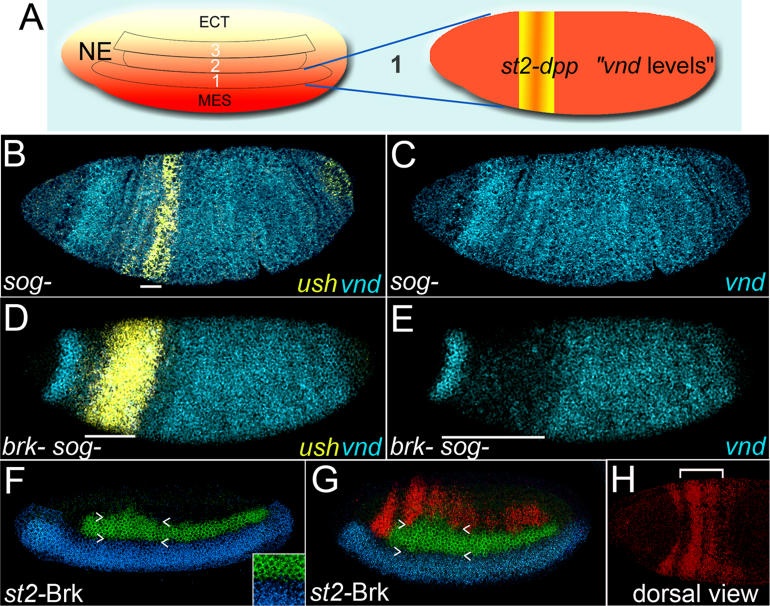
BMP Signaling Can Also Repress Expression of *vnd* (A) Scheme for generating ventro-lateralized embryos with a uniform level of Dorsal adjusted to that present in the ventral neuroectoderm (e.g., *vnd* expressing cells). These embryos were collected from *gd* ^7^ *sog* ^U2^/*gd* ^7^ *; Tl* ^3^/+ mothers (B and C) or *sog* ^Y506^ brk^m68^/FM7; *Tl* ^r4^/*Tl* ^r4^ mothers (D and E). (B and C) *ush* (yellow) and *vnd* (cyan) expression in a *sog*−*; st2-dpp* ventro-lateralized embryo. Note that while *ush* expression is induced in response to *dpp* expression in this embryo (bracket), the pattern of *vnd* expression remains unaffected. (D and E) *ush* (yellow) and *vnd* (cyan) expression in a *brk*− *sog*−*; st2-dpp* ventro-lateralized embryo. (D) The level and width of *ush* expression (bracket) is greater than in *sog*− single mutants (compare with [B]). (E) Note the broad domain of reduced *vnd* expression (bracket), which extends anterior to the *st2-dpp* expression domain. (F−H) Expression of *msh* (red), *ind* (green), and *vnd* (blue) in a *st2*-*brk* embryo that has a normal Dorsal gradient. (F) A mid-blastoderm stage embryo showing shifts in the dorsal and ventral borders of *ind* expression. The inset shows higher magnification of the *ind*/*vnd* border in the region of *st2-brk* expression, which is consistently shifted dorsally by 1−2 cells within the stripe of *brk* expression. This shift is most clearly revealed by a consistent flattening of what is normally a continuous arc in the *ind*/*vnd* border at the position of *st2*-*brk* expression. The inset also shows that *vnd* expression extends up to the *ind* border and that there is no gap between these gene expression domains. We quantitated the shift in the *ind*/*vnd* border in nine *st2-brk* and nine wild-type embryos by counting the number of *ind* negative cells above a line spanning the ventral border of *ind* expression in the head and abdomen comprising a zone four cells wide centered within the *st2* domain (or its approximate corresponding position in wild-type embryos). *ind* is better than *vnd* for performing this measurement since the ventral *ind* border is sharper (i.e., more all-or-none) than the dorsal *vnd* border. This analysis reveals that in *st2-brk* embryos there is an average of 5.4 ± 2.65 *ind* negative (*vnd* positive) cells above the line (which corresponds to an average shift of the *ind*/*vnd* border of 5.4/4 = 1.35 cells dorsally). In contrast, for control wild-type embryos we counted an average of 0.44 ± 0.88 cells above the line corresponding to an average of 0.11 cells. This represents a 10-fold difference between wild-type and *st2-brk* embryos, which is highly significant in a students' *t*-test (*p* < 0.0003). (G) Lateral view of a slightly older embryo than that shown in (F) showing a significant dorsal shift of the *msh*/*ind* border and a smaller shift of the *ind*/*vnd* border. The carets in (F and G) indicate the approximate trajectories of the *ind*/*vnd* and *msh/ind* borders in wild-type embryos. (H) Dorsal view of the same embryo shown in (G) revealing that *msh* expression expands to the dorsal midline.

Previous studies of the ventral-most neural identity gene, *vnd,* reported only a mild expansion of its expression domain in *dpp*− mutants [37], or no consistent effect [18]. We exploited our sensitive lateralized system to re-examine the BMP response of *vnd* in order to resolve these existing ambiguities (Figure 3). We expressed *st2*-*dpp* in embryos with uniform levels of Dorsal corresponding to the ventral neuroectoderm, which are sufficient to induce ubiquitous expression of *vnd* (Figure 3A). In such “ventro-lateralized” embryos, both *ind* and *msh* expression are absent, presumably due to repression by *vnd*. Elimination of *sog* function in these embryos resulted in activation of BMP signaling as judged by the localized activation of the epidermal marker *ush* (Figure 3B); however, *vnd* expression remained unaltered (Figure 3C). When we eliminated the function of both *sog* and the transcriptional repressor of BMP signaling, *brinker (brk),* we observed stronger and expanded expression of *ush* and potent repression of *vnd* in a broad zone centered over *st2-dpp* (Figure 3D and 3E). These results indicate that *vnd* is indeed sensitive to BMP-mediated repression and that Brk can block the repressive as well as activating [35] functions of BMP signaling. In analogy to what was observed in mid-lateralized embryos, it might have been expected that relief of Vnd repression in ventro-lateralized embryos would result in activation of *ind* in cells lacking *vnd* expression. However, we did not detect expression of either *ind* or *msh* in these embryos, even near the edges of the *vnd* repression domain (unpublished data). These data suggest that the high levels of Dpp signaling generated under these experimental conditions are sufficient to repress *vnd,* as well as *ind* and *msh*. Such strong BMP signaling, which is similar to that acting in the non-neural ectoderm of wild-type embryos, may obscure potential differences in the relative sensitivities of these genes to BMP-mediated repression by repressing expression of all neural genes. Although it remains to be determined what the relative sensitivity of *vnd* is to BMP repression (see Discussion), the fact that *vnd* is subject to such repression raises the possibility that Dpp might also regulate *vnd* expression along its dorsal border in wild-type embryos, despite the low levels of Dpp that diffuse into that region. Since the concentration of Dorsal is limiting with regard to activating *vnd* in cells along this border, these cells would be expected to be the most susceptible to BMP-mediated repression (see below).

### Dpp Activity Helps Establish Normal Borders of Neural Identity Gene Expression

Our analysis of BMP signaling in lateralized embryos showed that Dpp can regulate the expression of *ind* and *msh* in a dose-dependent fashion along the AP axis, and can also repress *vnd* expression. To test whether Dpp plays a similar dosage-sensitive role in the regulation of neural identity genes along the DV axis in the presence of an intact gradient of nuclear Dorsal, we devised an experiment to locally inhibit the response of neural genes to Dpp within the neuroectoderm of embryos with normal DV polarity. Because Brk can suppress BMP-mediated repression of *vnd* (Figure 3D and 3E), we reasoned that mis-expression of *brk* with the *eve-st2* enhancer might also relieve BMP repression of *ind* and *msh.* This localized expression of the *st2*-*brk* construct has the advantage of providing an internal comparison of gene expression domains within the same embryo. In embryos carrying the *st2*-*brk* construct, all three neural domains shifted dorsally at the site of *brk* over-expression (Figure 3F–3H). *msh* expression was de-repressed in a stripe dorsally (Figure 3H) as has been observed previously in *dpp*− mutants [8,18], and the border between *msh* and *ind* shifted dorsally by approximately 4–6 cells (Figure 3F and 3G). The dorsal shift in *ind* expression was observed prior to initiation of *msh* expression (Figure 3F), consistent with their normal ventral-to-dorsal sequence of activation [18]. In addition, we observed a modest but consistent dorsal shift of 1–2 cells in the *ind*/*vnd* border within the zone of *st2*-*brk* expression (Figure 3F, carets, see legend for quantification). The domains of *msh* and *ind* expression also shift in other situations where BMP signaling is altered in the context of an intact Dorsal gradient (see Figures S1 and S2), which reinforces the view that BMP signaling plays a role in determining the positions and extents of these expression domains in wild-type embryos.

The results described above indicate that graded Dpp activity normally plays an important role in establishing the position of the border between the *msh* and *ind* domains, and to a lesser degree influences the *ind*/*vnd* border, which forms 10–12 cells from the dorsal source of Dpp. The co-ordinate shifts in the borders of neural identity gene expression in *st2-brk* embryos are consistent with the known ventral-dominant chain of repression among *vnd, ind,* and *msh*. This analysis also provides additional support for *cis*-acting *vnd* sequences being sensitive to BMP repression and suggests that the dorsal border of *vnd* expression is normally determined by balancing the opposing influences of Dorsal activation [18,23,37] and BMP-mediated repression. We note that the dorsal expansion of *vnd* expression in *st2-brk* embryos does not necessarily imply that *vnd* is more sensitive to BMP-mediated repression than *ind* or *msh*, but instead that at limiting levels of Dorsal, even low levels of BMP signaling can exert a repressive effect on *vnd* expression.

### BMPs Act in a Dose-Dependent Fashion in Apolar Chick Neural Explants

Since Dpp was able to create elements of the neuroectodermal pattern in absence of other sources of DV polarity in *Drosophila* embryos, we wondered whether BMPs might have a similar organizing capacity in the vertebrate neural tube. Previous work has revealed that BMP signaling can act in a threshold-dependent fashion to pattern neural identity gene expression in vertebrates, however, the ability of BMPs to create pattern along the full DV axis in the absence of other graded cues has not been tested. As in *Drosophila*, a confounding problem in studying patterning in the vertebrate neural tube is to isolate the threshold-dependent effect of BMP signaling from that of a ventral gradient of Shh, which contributes to establishing the ordered expression of ventral and intermediate genes such as *Nkx2.2* and *Pax6* (Figure 1C) [30].

**Figure 4:**
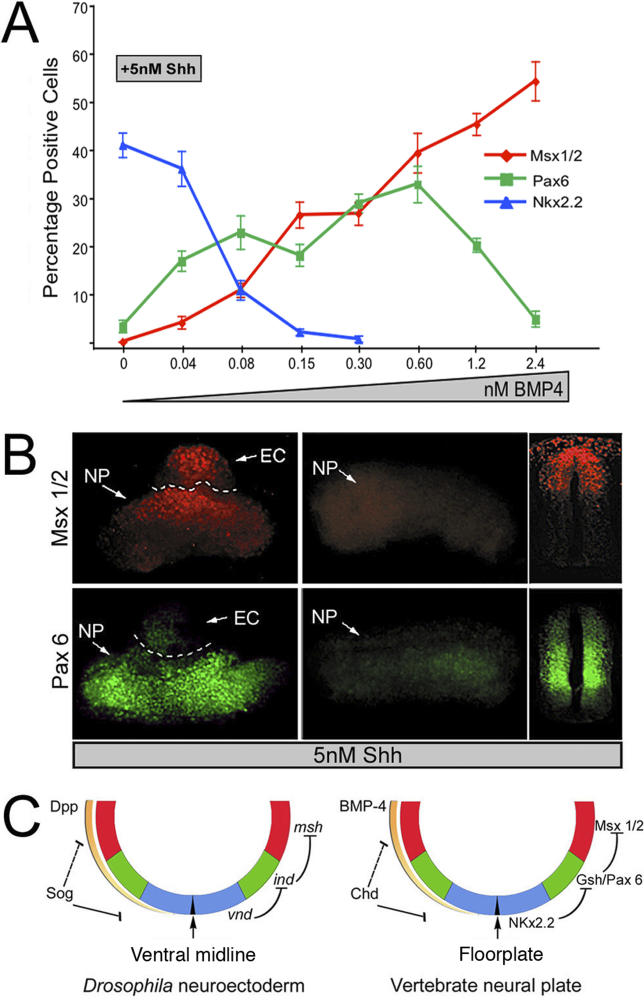
Dose-Dependent BMP-Mediated Repression in Neural Plate Explants (A) Chick intermediate neural plate explants were grown in 5 nM Shh and a range of BMP4 from 0 nM to 2.4 nM. Cells were stained with antibodies for Nkx2.2, Pax6, or Msx1/2 and number of positive cells per explant was counted and the percentage of positive cells was calculated and graphed. Error bars indicate standard error of the mean. The number of explants assayed was as follows, starting at 0 nM and ending at 2.4 nM BMP4: Nkx2.2 (25, 12, 15, 18, 12), Pax6 (18, 6, 18, 24, 30, 12, 24, 28), and Msx1/2 (12, 6, 16, 11, 17, 11, 17, 17). (B) Intermediate chick neural plate explants (NP) with (left) or without (right) BMP-expressing non-neural ectodermal tissue (EC) placed on the top edge, and cultured in the presence of 5 nM Shh. The fraction of Msx1/2-expressing cells is highest near the BMP source (top, left), and diminishes as a function of distance from the ectoderm. In a separate neural plate/ectoderm co-culture (bottom, left), Pax6 is expressed at a greater distance from the ectoderm, but not in nearby neural plate cells, which presumably express Msx1/2. These results mimic the relative expression domains of Msx1/2 and Pax6 in a stage 20 chick neural tube (far right). (C) Simplified summary model indicating the proposed similarities in BMP-mediated patterning of the vertebrate and invertebrate neuroectoderm. Two processes collaborate to establish the pattern of neural identity gene expression in *Drosophila* and vertebrates: graded BMP signaling preferentially represses expression of ventral neural identity genes (left), which then engage in a chain of ventral-dominant repression wherein more ventral genes prevail in repressing the expression of more dorsal genes (right). The indicated inhibition of *Msx1/2* by Pax6 remains hypothetical. Not indicated on this scheme are additional levels of cross-inhibition (e.g., Vnd inhibition of *msh,* late Ind repression of *vnd,* and Pax6 repression of *Nkx2.2*) [9,10,16,21,61,62], which are likely to help sharpen and refine the pattern created by the core mechanism of threshold-dependent BMP repression coupled to ventral dominance.

To isolate the patterning effect of BMPs from that of Hedgehog (Hh) we used a strategy similar to that devised in *Drosophila* for creating a field of cells with uniform DV identity. Naive chick neural plate explants were treated with 5 nM Shh, which induces ventral cell fates (Figure 4). In this ventralized background, we added BMPs over a range of concentrations and determined the percentage of cells expressing Nkx2.2, Pax6, and Msx1/2 (Figure 4A). We assayed Pax6 expression in these experiments rather than the *ind* ortholog *Gsh,* since *Gsh* expression is initiated after neurogenesis in the chick, and because Pax6 may fulfill a similar role in specifying cell fates in the intermediate region of the neural tube [17]. In the absence of added BMPs, the explants only expressed the ventral marker Nkx2.2, which represses Pax6 expression [21]. As the BMP4 concentration increased, the percentage of cells expressing Nkx2.2 declined at the same time the fraction of cells expressing intermediate (Pax6) and dorsal (Msx1/2) markers increased (Figure 4A). Concomitant with the initial decrease in Nkx2.2-expressing cells, we observed an increase in the number of motor neurons, which differentiate in the ventral-intermediate neural tube, as measured by expression of Isl1/2 (unpublished data). At the highest levels of BMP4 tested, the great majority of cells expressed only the dorsal-most marker Msx1/2. In addition, a significant number of neural crest cells, which derive from the dorsal-most region of the neural tube, migrated from the explants as revealed by HNK-1 staining (unpublished data). These results demonstrate that normal patterning in the neural tube can be recapitulated in ventralized neural tissue by altering BMP4 levels alone. Although we have not observed repression of Msx1/2 at the highest dose of BMP tested, in zebrafish it has been suggested that high levels of BMP signaling may repress these genes [43]. It is unclear whether these differences reflect experimental design or inherent differences between organisms.

We further tested the organizing activity of BMPs by asking whether an ectodermal graft, which acts as a localized source of BMPs, could induce patterned expression of neural identity genes in chick neural plate explants. We placed ventralized Nkx2.2-expressing explants into contact with BMP-expressing ectodermal cells along one edge of the explant (Figure 4B). This experimental scheme, which is similar in concept to expressing a stripe of *dpp* in *sog*− lateralized *Drosophila* embryos, resulted in loss of Nkx2.2 expression and induction of concentric domains of Msx1/2 and Pax6 expression. In these grafted co-cultures, Msx1/2 was expressed in a band of cells in the neural plate explant closest to the ectodermal tissue (Figure 4B, top left), while strong Pax6 expression was excluded from cells near the source of BMPs, but was observed in a broad zone further away from the graft (Figure 4B, lower left). These results reveal that in the absence of other DV patterning cues, BMPs can diffuse over a long range into the neural plate explant and act in a dosage-sensitive fashion to pattern expression of neural identity genes in the same order as in the endogenous neural tube. We conclude that diffusion of BMPs alone can establish pattern along the full DV axis of the vertebrate neural tube.

## Discussion

It is generally assumed that divergent upstream regulatory processes activate orthologous sets of neural identity genes in the same DV order [7,18]. This model is analogous to that for convergent regulation of *HOX* genes in a conserved order along the AP axis [22]. Two primary considerations have lead to the view that the conserved DV pattern of neural gene expression may have evolved independently in these two lineages, rather than reflecting a common ancestral state. First, until the current study, there has been no evidence that BMPs act in a dose-dependent fashion in *Drosophila*. Second, in vertebrates, where BMPs have been shown to act in a dose-dependent fashion to pattern the neural tube [33,34,43–49], a common view is that they promote expression of neural identity genes such as *Msx1/2* in dorsal regions of the neural tube [33]. In flies, however, BMPs have only been observed to repress expression of neural genes, suggesting that the underlying mechanisms of neural gene regulation by BMPs may be fundamentally different [7,18].

An alternative hypothesis for neuroectodermal patterning that reconciles existing data is that BMPs act by a mechanism analogous to that operating in *Drosophila,* namely by preferentially repressing expression of more ventral neural identity genes. An attractive feature of this simple unified model is that the mechanism of BMP action is the same during both neural induction and neuroectodermal patterning. The only difference is that during the latter process, BMPs diffusing into the neuroectoderm are present at much lower levels, with the effect that the repression of neural gene expression becomes dependent on BMP dosage.

### Dpp Mediates Dose-Dependent Repression of Neural Identity Genes in *Drosophila*

In this study, we present two lines of evidence that BMPs act in a dosage-dependent fashion to help pattern the *Drosophila* neuroectoderm. First, we find that a localized source of Dpp is capable of creating patterned expression of *msh* and *ind* in lateralized embryos with no other known source of DV patterning. Second, we show that localized inhibition of BMP signaling in embryos with a normal Dorsal gradient shift all three borders of neural identity gene expression dorsally. The simplest explanation for these results is that BMP signaling represses neural gene expression in the neuroectoderm, as it does earlier during neural induction. However, in contrast to neural induction, where the high levels of BMP signaling present in the epidermal ectoderm completely abolish the expression of all neural genes, the considerably lower levels of BMPs present in the neuroectoderm act in a threshold-dependent fashion to create separate domains of neural gene expression. Neural identity genes expressed more ventrally are most susceptible to this graded BMP repression, wheras those expressed in more dorsal positions are progressively less sensitive. Ventral-dominant cross-repression among neural identity genes, which is a well-established phenomenon in *Drosophila* akin to posterior dominance among *HOX* genes along the AP axis, helps refine the pattern into mutually exclusive domains of neural identity gene expression. These findings demonstrate that as in vertebrate embryos, BMPs act in a dosage-sensitive fashion to contribute to neuroectodermal patterning.

This model of threshold-dependent BMP-mediated repression is also consistent with the previous observation that Screw, another BMP that acts synergistically with Dpp in embryos, is required to repress *msh* expression in dorsal cells (C. M. Mizutani, unpublished data). Interestingly, other neural genes such as those of the Achaete-Scute complex are de-repressed in *dpp*− embryos, but not in *scw*− mutants [27], providing a further indication that higher levels of BMP signaling are required to repress *msh* expression than other neural genes.

### A Conserved Ancestral Role of BMPs in Patterning the Neuroectoderm?

The results we obtained in lateralized chick neural plate explants are strikingly similar to those observed in lateralized *Drosophila* embryos. We observed that increasing concentrations of BMPs progressively favors the expression of dorsal over ventral neural identity genes. We also found that a graft of BMP-expressing cells could organize graded spatial expression of dorsal and lateral markers in lateralized neural plate explants in much the same way that a localized source of Dpp can create pattern in the context of uniform levels of Dorsal in lateralized *Drosophila* embryos. These findings are consistent with prior evidence that BMPs act in a dose-dependent fashion to pattern gene expression in dorsal and lateral regions of the vertebrate neural tube [33,34,43–49]. Previous studies also provided evidence that BMPs extend their influence into ventral regions of the neural tube and in conjunction with graded levels of Shh can cause a ventral-to-dorsal shift in the identities of neural progenitor cells [32,50–52]. In our current study we extend these findings by showing that BMPs can act in the absence of other patterning cues to mediate normal patterning of the neural tube, including the induction of motorneuron-specific markers. In addition, we show that a localized source of BMPs from the ectoderm is capable of establishing a graded response in apolar neural plate explants over long range. These data, in principle, could be explained by a model similar to that proposed above for *Drosophila,* in which BMP signaling acts by repressing neural gene expression (Figure 4C).

The view that BMPs may repress neural gene expression in vertebrates runs contrary to the currently favored model in which BMPs activate expression of target genes [33,34]. Evidence cited in favor of the “activation” model include the fact that over-expression of BMPs in *Xenopus* can induce Msx1 expression in the presence of cyclohexamide, suggesting a direct activation of *Msx1* by BMPs [53]. Also, Msx1 and Msx2 promoter elements have been identified that mediate BMP-dependent activation of reporter gene constructs [54–56].

There are important caveats, however, to the interpretation that BMPs activate expression of *Msx* genes in the neural ectoderm, which stem from the fact that the *Msx1/2* genes are expressed in complex patterns that include non-neural as well as neural components. For example, in *Xenopus* and zebrafish embryos, Msx1 is expressed in two stripes of cells, one in ventral ectodermal cells and the other dorsally at the border between the ectoderm and neural ectoderm. Since the strongest expression is detected ventrally, it was not possible in the original experiments to distinguish between *Msx1* activation by BMPs in ventral versus dorsal cells [53]. The observation that the dorsal domain of *Msx1* expression is graded ventrally and is lost in more central regions of the non-neural ectoderm [53] suggests that high levels of BMP signaling found in those regions may actually play a role in repressing *Msx1/2* expression. Consistent with this possibility, when BMP signaling was blocked selectively in cells giving rise to the dorsal *Msx1* domain in zebrafish, *Msx1* expression expanded [43]. This finding suggests that the *Msx1/2* and *msh* genes may be similarly regulated by BMPs in both vertebrates and flies, wherein high levels of BMP signaling repress rather than activate expression of these orthologous genes. Interestingly, BMPs have also been proposed to repress the expression of *Pax6* in the forebrain [57–59] and to potently repress the lateral expression of *Dbx1/2* genes in the neural tube [60].

It is also noteworthy that while minimal *Msx* enhancer elements have been described to respond positively to BMP signaling and drive parts of the endogenous gene expression profiles, none of these elements drive expression in the dorsal region of the neural plate [54–56]. Thus, it remains to be determined whether *Msx1/2* genes are activated directly by BMP signaling in the dorsal neural tube or indirectly by relieving cross-inhibition mediated by more ventrally expressed transcription factors such as Gsh, Pax6, or Dbx1,2. Further analysis of regulatory elements directing faithful expression of *Msx1/2* and other neural identity genes in the neuroectoderm will be required to address this question.

Based on the considerations raised above, we favor a parsimonious model in which BMPs function similarly during patterning of the neuroectoderm in vertebrates and invertebrates. According to this hypothesis, an ancestral cascade of sensitivity of neural identity genes to BMP-mediated repression has been preserved intact in vertebrates whereas in *Drosophila*, only the two dorsal-most genes *(ind* and *msh)* may have remained differentially sensitive to BMP repression. Although the relative sensitivity of *vnd* and *ind* to BMP repression in *Drosophila* is unresolved, it is nonetheless apparent that along the dorsal border of *vnd* expression, where levels of the Dorsal activator are limiting, BMP signaling plays a role in opposing Dorsal activation of *vnd*. In the absence of BMP repression, *vnd* expression expands dorsally 1–2 cells, where it presumably represses *ind* expression by virtue of ventral dominance. As mentioned above, it may also be the case that in vertebrates the *Msx1/2* genes have become relatively less sensitive to BMP repression since *Msx1* is expressed in neural crest forming cells of the non-neural ectoderm immediately adjacent to the neural plate [53].

### Mechanisms for Refining the Graded BMP Response

The graded positional information created by BMP signaling in the neuroectoderm is subsequently refined by cross-inhibitory interactions among neural identity genes. The result of these interactions is to segregate gene expression patterns into non-overlapping adjacent territories. In *Drosophila,* cross-regulatory interactions follow a strict ventral-dominant hierarchy in which ventral genes repress more dorsal ones. In vertebrates, neural identity genes also cross-inhibit each other [21,61,62], although it has not been resolved whether these interactions are biased along the DV axis. There is some evidence that also favors ventral dominance in vertebrates, however. For example, Nkx2.2, can inhibit the ectopic expression of Pax6 in ventral neural tube cells in which Hh signaling has been blocked (e.g., see Figure S3). In addition, in *Xenopus tropicalis,* it has recently been observed that mis-expression of the *Gsh2* gene in the neural plate represses dorsal expression of *Msx1*, but not ventral expression of *Nkx6.1* (H. Isaacs, unpublished data), consistent with these transcription factors acting in a ventral-dominant fashion as they do in flies. While additional studies will be required to establish whether mutual cross-inhibition among vertebrate neural identity genes follows a clear ventral hierarchical order, such interactions, even if symmetrical, would nonetheless act to resolve biases in gene expression created by a graded response to BMPs. Consistent with a conserved function of BMP signaling in the neuroectoderm, we find that BMPs act in a dose-dependent fashion in lateralized apolar chick neural plate explants to create patterned expression of neural identity genes in much the same way they do in lateralized *Drosophila* embryos.

Another mechanism for shaping the BMP activity and refining the neural domains may be ventral expression of BMP antagonists. In *Drosophila,* Sog initially occupies the entire neuroectoderm (Figure 1D) and then fades progressively from dorsal cells as *msh* expression is initiated (Figure 1D and 1E) [38,39]. This trend continues until *sog* expression is confined to the ventral midline. As *sog* expression fades dorsally, it may create a broader domain of elevated BMP signaling sufficient to repress *ind* and to permit *msh* activation. In *sog*− lateralized embryos, the separation of *msh* and *ind* domains is less defined than in wild-type embryos, which may result from the lack of ventral Sog and Brk gradients that normally contribute to refining these expression domains. In addition, some cells co-express both *msh* and *ind*, albeit few cells express these genes at comparable levels. It may be that a gradient of Dorsal is required to establish full ventral dominance under these conditions. Alternatively, ventral dominance may normally depend in part on the progressive temporal activation of neural identity genes in a ventral-to-dorsal pattern, which may help more ventral genes consolidate their expression domains. In vertebrates, BMP antagonists such as Chd and Noggin emanating from the notochord [32,63] (Figure 1C) may likewise help sharpen the BMP gradient ventrally.

### Secondary Evolution of Species-Specific DV Patterning Systems

One marked difference in the integrated systems acting to pattern the neuroectoderm in flies and vertebrates is that distinct pathways have evolved in addition to the proposed conserved core of BMP-mediated patterning. While BMPs appear to play a major if not predominant role in specifying cell fates in dorsal regions of the fly and vertebrate neuroectoderm, the relative importance of these signaling systems is reversed in ventral regions where Dorsal and Shh play primary patterning roles in flies and vertebrates respectively. In the lateral column, a combination of BMPs and ventral patterning cues (Dorsal and Shh) act in concert to determine the position and extent of gene expression.

The deployment of Shh in patterning the ventral neural tube appears to be a vertebrate innovation since no ventral source of Hh signaling is observed in *Drosophila* [7] or in basal arthropods such as millipedes [64]. Graded Hh signaling may have arisen during the course of chordate/vertebrate evolution to supplement a pre-existing long range BMP signaling system. The secondary recruitment of Hh signaling to DV patterning is further supported by the finding that in the complete absence of Hh signaling, ventral and intermediate neural cell types still form. Interestingly, Nkx2.2 expression is not observed in such embryos, which may be due to the loss of notochord-derived BMP antagonists [65–69]. It has been suggested that the restoration of ventral pattern in the absence of Hh signaling could be provided by the remaining BMP gradient [19]. Furthermore, the notochord-derived BMP antagonists Noggin and Chd can act synergistically with Shh in the neural tube to promote ventral cell fates, including Nkx2.2 expression [31,32]. These observations are consistent with evidence that BMPs exert an influence along the entire dorsal-ventral axis of neural tube [34,46].

The NFκB-related transcription factor Dorsal may also have been co-opted to assume a prominent role in patterning the ventral portion of the *Drosophila* neuroectoderm, leading eventually to a reduction in the peak sensitivity of *vnd* to BMP repression. In addition, Dorsal is likely to act in conjunction with its target genes Sog and Brk to refine the *ind* and *msh* expression domains. Perhaps these later lineage-specific adaptations evolved to help sharpen and reinforce the effect of BMP signaling, particularly at the low end of the concentration gradient. One potential evolutionary pressure in creating a secondary ventral organizer may have been an increase in body size. It has been proposed that early bilateralia may have been only a few millimeters or less in size (for reviews see [70,71]). In organisms of such small dimension, a single morphogen may have formed a sufficiently steep concentration gradient over short distances to reliably subdivide the entire DV axis into discrete domains. As larger organisms evolved during Precambrian radiations, however, a single morphogen gradient may no longer have been sufficient to provide the same detailed patterning information. Although, as mentioned above, BMPs are apparently able to diffuse substantial distances into ventral regions of the vertebrate neural tube, such gradients may be too flat in distant regions to produce sharp patterns. Indeed, in animals lacking all Hh signaling, while ventral genes are expressed in approximately normal domains, these expression patterns are not as sharp as in wild-type embryos.

In view of the fact that BMP signaling plays an important role in patterning the non-neural ectoderm as well as the neuroectoderm of *Drosophila* and vertebrates, we propose that graded BMP signaling created by opposing sources of BMPs and BMP-inhibitors such as Sog/Chd (Figure 4C) may once have been sufficient to establish ordered domains of gene expression along the full DV axis of the ectoderm. According to this model, high BMP levels in the non-neural ectoderm were sufficient to suppress expression of all neural genes. Diffusion of Sog/Chd into this region created graded high-level BMP signaling, which lead to threshold-dependent activation of BMP target genes and subsequent partitioning of cells into discrete territories. In the neuroectoderm, where graded levels of BMPs became limiting, threshold-dependent repression of gene expression provided the spatial information for subdividing that region into three primary domains. Thus, by a combination of threshold-dependent activation and repression of target genes in epidermal versus neural regions of the embryo, BMPs may have once organized the entire DV axis of ancestral metazoa.

## Materials and Methods

### *Drosophila* stocks and genetic crosses.

The following stocks were used in this study: Df(2L)DTD48/CyO23 (deficiency for *dpp*), *yw; st2-dpp* (provided by H. Ashe), *w;* FlpT1, and *w; st2-*FRT-stop-FRT-*brk* (provided by S. Small). We briefly provide a summary of the maternal mutations used to generate lateralized embryos with uniform levels of Dorsal. The *Tl^3^* mutation encodes a partially activated form of Toll receptor capable of transducing the signal for Dorsal transport into all nuclei independently from the ligand Spätzle. *gastrulation defective* is an upstream component of the Toll pathway required for the production of Spätzle. Combining *gd* ^7^ *,* a null allele of *gastrulation defective,* with *Tl* ^3^/+ in females leads to the production of embryos with uniform levels of Dorsal, since the remaining wild type copy of Toll is no longer activated by ligand. The levels of Dorsal in *gd* ^7^ *; Tl* ^3^/+ embryos correspond to those specifying the ventral neuroectoderm (e.g., expressing *vnd* uniformly). Further reduction of Dorsal levels in *gd* ^7^ *; dl^1^*/+; *Tl* ^3^/+ females results in mid-lateralized embryos (e.g., expressing *ind* uniformly). An alternative way to generate ventro-lateralized embryos is to collect them from mothers homozygous from the weakly activated *Tl* ^r4^ allele. In some experiments it was also necessary to eliminate zygotic function of *sog* or *sog* and *brk* in embryos using respectively the *sog* ^U2^ or *sog* ^y506^ *brk* ^m68^ alleles. The maternal genotypes described in text were generated according to the following crosses (recessive markers omitted for clarity): to generate *gd* ^7^ *sog* ^U2^/ *gd* ^7^ *; dl* ^1^/+*; Tl^3^*/+ females for producing mid-lateralized *sog*− male embryos, *gd* ^7^ *sog* ^U2^/FM7; *dl* ^1^ /CyO females were crossed to *gd* ^7^/Y; *Tl^3^/*TM3, *Sb* males (Figure 1D, Figure 2); to generate *gd* ^7^ *sog* ^U2^/ *gd* ^7^ *; Tl^3^*/+ females for producing ventro-lateralized *sog*− male embryos, *gd* ^7^ *sog* ^U2^/FM7 females were crossed to *gd* ^7^/Y; *Tl* ^3^/+ males (Figure 3B and 3C), and to generate *sog* ^y506^ *brk* ^m68^/ FM7; *Tl* ^r4^/ *Tl* ^r4^ females for producing ventro-lateralized *sog*− *brk*− male embryos used in the experiments described in Figure 3D and 3E, *sog* ^y506^ *brk* ^m68^/FM7; *Tl* ^r4^/*Tl* ^r4^ females were selected from a *sog* ^y506^ *brk* ^m68^/FM7; *Tl* ^r4^/TM3, *Sb* stock. Females collected from crosses described above were crossed to *w* males (control) or to *yw; st2-dpp* males (experimental), and embryos lacking *sog* mRNA expression were analyzed for other gene expression patterns. Other standard mutant stocks were obtained from the Bloomington Stock Center (Bloomington, Indiana, United States) and are described in Lindsley and Zimm [72] or Flybase (http://flybase.bio.indiana.edu).

### Immunofluorescence and in situ hybridization.

Fluorescent multiplex in situ hybridization methods used to detect multiple RNA transcripts are described in detail in Kosman et al [42]. For double protein detection and in situ staining, fixed embryos were treated with acetone (10 min at −20° C), hybridized with probes, followed by immunostaining with rabbit anti-phosphoMAD (1:2,000, a gift from P. ten Dijke) and antibodies were used to detect the RNA labeled probes. Detection of primary antibodies was performed either with secondary antibodies labeled with Alexa Fluor dyes (used at 1:500, Molecular Probes, Eugene, Oregon, United States) or using the Zenon kit (Molecular Probes). A list of primary and secondary antibodies used in these experiments is available in the online supplementary material of Kosman et al [42]. Images of fluorescently labeled embryos were acquired on a Leica SP2-AOBS (Leica Microsystems, Wetzlar, Germany) scanning confocal microscope with 20× and/or 40× objective lenses.

### Culture and immunofluorescence staining of chick neural plate explants.

Chick neural plate explants were cultured in collagen pillows as described in [73] for 28 h with 5 nM Shh prepared as described in [74] and in the absence or presence of varying concentrations of human recombinant BMP4 (R&D Systems, Minneapolis, Minnesota, United States). In co-culture experiments, intermediate chick neural plate explants were cultured for 28 h in collagen pillows with or without non-neural ectoderm adjacent to the neural plate explants. 5 nM Shh was added at the beginning of the co-cultures. Dissection efficiency and the border between intermediate neural plate explants and ectoderm explants were examined with NCAM (labels neural plate cells) and chick L-Cadherin (labels ectodermal cells) antibodies. Staining on explants was carried out according to [74]. For visualization, explants were cleared in 80% glycerol in PBS and then mounted in Biomeda's Gel Mount (Biomeda, Foster City, California, United States). Fluorescent images were captured using a Nikon (Melville, New York, United States) Microphot SA microscope, Diagnostic Instruments (Sterling Heights, Michigan, United States) Spot RT Slider, and Spot 3.2 software.

## Supporting Information

Figure S1 BMP Represses *ind* at a Longer Range than *msh*
A *sog*−*; st2-dpp* embryo with an intact Dorsal gradient stained for *dpp* (blue), *ind* (green), and *msh* (red). *ind* is repressed in a broad domain (bars) from the source of *dpp* expression. In regions of strong *ind* repression, *msh* expression invades ventrally into the r2 domain (arrows).
(4.0 MB TIF)

Figure S2 Differential Sensitivities of *ind* and *msh* Regulation to BMP Repression A *sog-brk*−; Dp(2;2) DTD48 embryo which has normal DV polarity and high levels of Dpp within the neuroectoderm. Low levels of *msh* expression (red) can be detected within the intermediate neuroectoderm domain, where *ind* expression (green) is largely lost.
(6.2 MB TIF)

Figure S3 Ventral Expansion of Pax6 in Response to Inhibition of Shh Signaling is Mediated by Repression of Nkx2.2 (A) Mis-expression of the Gli3 repressor (Gli3R) causes cell autonomous expression of Pax6 as well as repression of Nkx2.2 (unpublished data).
(B) Co-expression of Nkx2.2 with Gli3R reverses Pax6 activation by Gli3R.
(8.2 MB TIF)

## Abbreviations

AP: anterior-posterior
BMP: bone morphogenetic protein
Brk: Brinker
Chd: Chordin
Dpp: Decapentaplegic
DV: dorsal-ventral
Hh: Hedgehog
*ind*: *intermediate nervous system defective*
*msh*: *muscle specific homeobox*
pMAD: phosphorylated form of Mothers against dpp
Shh: Sonic Hedgehog
Sog: Short gastrulation
*st2-dpp,*: *even-skipped* stripe 2 enhancer driving expression of *dpp*
*ush*: *u-shaped*
*vnd*: *ventral nervous system defective*

## References

[pbio-0040313-b001] Freeman M, Gurdon JB (2002). Regulatory principles of developmental signaling. Annu Rev Cell Dev Biol.

[pbio-0040313-b002] Tabata T, Takei Y (2004). Morphogens, their identification and regulation. Development.

[pbio-0040313-b003] Bier E (1997). Anti-neural-inhibition: A conserved mechanism for neural induction. Cell.

[pbio-0040313-b004] Eldar A, Dorfman R, Weiss D, Ashe H, Shilo BZ (2002). Robustness of the BMP morphogen gradient in *Drosophila* embryonic patterning. Nature.

[pbio-0040313-b005] Shimmi O, Umulis D, Othmer H, O'Connor MB (2005). Facilitated transport of a Dpp/Scw heterodimer by Sog/Tsg leads to robust patterning of the *Drosophila* blastoderm embryo. Cell.

[pbio-0040313-b006] Mizutani CM, Nie Q, Wan FY, Zhang YT, Vilmos P (2005). Formation of the BMP activity gradient in the *Drosophila* embryo. Dev Cell.

[pbio-0040313-b007] Arendt D, Nubler-Jung K (1999). Comparison of early nerve cord development in insects and vertebrates. Development.

[pbio-0040313-b008] D'Alessio M, Frasch M (1996). *msh* may play a conserved role in dorsoventral patterning of the neuroectoderm and mesoderm. Mech Dev.

[pbio-0040313-b009] Cowden J, Levine M (2003). Ventral dominance governs sequential patterns of gene expression across the dorsal-ventral axis of the neuroectoderm in the *Drosophila* embryo. Dev Biol.

[pbio-0040313-b010] Cornell RA, Ohlen TV (2000). *vnd*/*Nkx, ind*/*Gsh,* and *msh*/*Msx:* Conserved regulators of dorsoventral neural patterning?. Curr Opin Neurobiol.

[pbio-0040313-b011] Jimenez F, Martin-Morris LE, Velasco L, Chu H, Sierra J (1995). *vnd,* a gene required for early neurogenesis of *Drosophila,* encodes a homeodomain protein. Embo J.

[pbio-0040313-b012] McDonald JA, Holbrook S, Isshiki T, Weiss J, Doe CQ (1998). Dorsoventral patterning in the *Drosophila* central nervous system: The *vnd* homeobox gene specifies ventral column identity. Genes Dev.

[pbio-0040313-b013] Skeath JB, Panganiban GF, Carroll SB (1994). The *ventral nervous system defective* gene controls proneural gene expression at two distinct steps during neuroblast formation in *Drosophila*. Development.

[pbio-0040313-b014] Isshiki T, Takeichi M, Nose A (1997). The role of the *msh* homeobox gene during *Drosophila* neurogenesis: Implication for the dorsoventral specification of the neuroectoderm. Development.

[pbio-0040313-b015] Chu H, Parras C, White K, Jimenez F (1998). Formation and specification of ventral neuroblasts is controlled by *vnd* in *Drosophila* neurogenesis. Genes Dev.

[pbio-0040313-b016] Weiss JB, Von Ohlen T, Mellerick DM, Dressler G, Doe CQ (1998). Dorsoventral patterning in the *Drosophila* central nervous system: The *intermediate neuroblasts defective* homeobox gene specifies intermediate column identity. Genes Dev.

[pbio-0040313-b017] Briscoe J, Pierani A, Jessell TM, Ericson J (2000). A homeodomain protein code specifies progenitor cell identity and neuronal fate in the ventral neural tube. Cell.

[pbio-0040313-b018] von Ohlen T, Doe CQ (2000). Convergence of *dorsal, dpp,* and *egfr* signaling pathways subdivides the *Drosophila* neuroectoderm into three dorsal-ventral columns. Dev Biol.

[pbio-0040313-b019] Jacob J, Briscoe J (2003). Gli proteins and the control of spinal-cord patterning. EMBO Rep.

[pbio-0040313-b020] McMahon AP (2000). Neural patterning: The role of *Nkx* genes in the ventral spinal cord. Genes Dev.

[pbio-0040313-b021] Muhr J, Andersson E, Persson M, Jessell TM, Ericson J (2001). Groucho-mediated transcriptional repression establishes progenitor cell pattern and neuronal fate in the ventral neural tube. Cell.

[pbio-0040313-b022] Gellon G, McGinnis W (1998). Shaping animal body plans in development and evolution by modulation of *HOX* expression patterns. Bioessays.

[pbio-0040313-b023] Stathopoulos A, Levine M (2002). Dorsal gradient networks in the *Drosophila* embryo. Dev Biol.

[pbio-0040313-b024] Markstein M, Markstein P, Markstein V, Levine MS (2002). Genome-wide analysis of clustered Dorsal binding sites identifies putative target genes in the *Drosophila* embryo. Proc Natl Acad Sci U S A.

[pbio-0040313-b025] Stathopoulos A, Levine M (2005). Localized repressors delineate the neurogenic ectoderm in the early *Drosophila* embryo. Dev Biol.

[pbio-0040313-b026] Ruiz i Altaba A, Nguyen V, Palma V (2003). The emergent design of the neural tube: Prepattern, SHH morphogen, and GLI code. Curr Opin Genet Dev.

[pbio-0040313-b027] Biehs B, Francois V, Bier E (1996). The *Drosophila short gastrulation* gene prevents Dpp from autoactivating and suppressing neurogenesis in the neuroectoderm. Genes Dev.

[pbio-0040313-b028] Kishimoto Y, Lee KH, Zon L, Hammerschmidt M, Schulte-Merker S (1997). The molecular nature of zebrafish *swirl*: BMP2 function is essential during early dorsoventral patterning. Development.

[pbio-0040313-b029] Oelgeschlager M, Kuroda H, Reversade B, De Robertis EM (2003). Chordin is required for the Spemann organizer transplantation phenomenon in *Xenopus* embryos. Dev Cell.

[pbio-0040313-b030] Patten I, Placzek M (2002). Opponent activities of Shh and BMP signaling during floor plate induction in vivo. Curr Biol.

[pbio-0040313-b031] McMahon JA, Takada S, Zimmerman LB, Fan CM, Harland RM (1998). Noggin-mediated antagonism of BMP signaling is required for growth and patterning of the neural tube and somite. Genes Dev.

[pbio-0040313-b032] Liem KF, Jessell TM, Briscoe J (2000). Regulation of the neural patterning activity of sonic hedgehog by secreted BMP inhibitors expressed by notochord and somites. Development.

[pbio-0040313-b033] Lee KJ, Jessell TM (1999). The specification of dorsal cell fates in the vertebrate central nervous system. Annu Rev Neurosci.

[pbio-0040313-b034] Nguyen VH, Trout J, Connors SA, Andermann P, Weinberg E (2000). Dorsal and intermediate neuronal cell types of the spinal cord are established by a BMP signaling pathway. Development.

[pbio-0040313-b035] Jazwinska A, Rushlow C, Roth S (1999). The role of *brinker* in mediating the graded response to Dpp in early *Drosophila* embryos. Development.

[pbio-0040313-b036] Skeath JB, Panganiban G, Selegue J, Carroll SB (1992). Gene regulation in two dimensions: The proneural *achaete* and *scute* genes are controlled by combinations of axis-patterning genes through a common intergenic control region. Genes Dev.

[pbio-0040313-b037] Mellerick DM, Nirenberg M (1995). Dorsal-ventral patterning genes restrict *NK-2* homeobox gene expression to the ventral half of the central nervous system of *Drosophila* embryos. Dev Biol.

[pbio-0040313-b038] Francois V, Solloway M, O'Neill JW, Emery J, Bier E (1994). Dorsal-ventral patterning of the *Drosophila* embryo depends on a putative negative growth factor encoded by the *short gastrulation* gene. Genes Dev.

[pbio-0040313-b039] Srinivasan S, Rashka KE, Bier E (2002). Creation of a Sog morphogen gradient in the *Drosophila* embryo. Dev Cell.

[pbio-0040313-b040] Araujo H, Bier E (2000). *sog* and *dpp* exert opposing maternal functions to modify Toll signaling and pattern the dorsoventral axis of the *Drosophila* embryo. Development.

[pbio-0040313-b041] Rusch J, Levine M (1994). Regulation of the dorsal morphogen by the Toll and Torso signaling pathways: A receptor tyrosine kinase selectively masks transcriptional repression. Genes Dev.

[pbio-0040313-b042] Kosman D, Mizutani CM, Lemons D, Cox WG, McGinnis W (2004). Multiplex detection of RNA expression in *Drosophila* embryos. Science.

[pbio-0040313-b043] Tribulo C, Aybar MJ, Nguyen VH, Mullins MC, Mayor R (2003). Regulation of Msx genes by a Bmp gradient is essential for neural crest specification. Development.

[pbio-0040313-b044] Marchant L, Linker C, Ruiz P, Guerrero N, Mayor R (1998). The inductive properties of mesoderm suggest that the neural crest cells are specified by a BMP gradient. Dev Biol.

[pbio-0040313-b045] LaBonne C, Bronner-Fraser M (1998). Neural crest induction in *Xenopus*: Evidence for a two-signal model. Development.

[pbio-0040313-b046] Barth KA, Kishimoto Y, Rohr KB, Seydler C, Schulte-Merker S (1999). Bmp activity establishes a gradient of positional information throughout the entire neural plate. Development.

[pbio-0040313-b047] Neave B, Holder N, Patient R (1997). A graded response to BMP-4 spatially coordinates patterning of the mesoderm and ectoderm in the zebrafish. Mech Dev.

[pbio-0040313-b048] Wilson PA, Lagna G, Suzuki A, Hemmati-Brivanlou A (1997). Concentration-dependent patterning of the *Xenopus* ectoderm by BMP4 and its signal transducer Smad1. Development.

[pbio-0040313-b049] Timmer JR, Wang C, Niswander L (2002). BMP signaling patterns the dorsal and intermediate neural tube via regulation of homeobox and helix-loop-helix transcription factors. Development.

[pbio-0040313-b050] Arkell R, Beddington RS (1997). BMP-7 influences pattern and growth of the developing hindbrain of mouse embryos. Development.

[pbio-0040313-b051] Basler K, Edlund T, Jessell TM, Yamada T (1993). Control of cell pattern in the neural tube: Regulation of cell differentiation by Dorsalin-1, a novel TGF beta family member. Cell.

[pbio-0040313-b052] Liem KF, Tremml G, Roelink H, Jessell TM (1995). Dorsal differentiation of neural plate cells induced by BMP-mediated signals from epidermal ectoderm. Cell.

[pbio-0040313-b053] Suzuki A, Ueno N, Hemmati-Brivanlou A (1997). *Xenopus* msx1 mediates epidermal induction and neural inhibition by BMP4. Development.

[pbio-0040313-b054] Alvarez Martinez CE, Binato R, Gonzalez S, Pereira M, Robert B (2002). Characterization of a Smad motif similar to Drosophila mad in the mouse Msx 1 promoter. Biochem Biophys Res Commun.

[pbio-0040313-b055] Brugger SM, Merrill AE, Torres-Vazquez J, Wu N, Ting MC (2004). A phylogenetically conserved *cis*-regulatory module in the Msx2 promoter is sufficient for BMP-dependent transcription in murine and *Drosophila* embryos. Development.

[pbio-0040313-b056] MacKenzie A, Purdie L, Davidson D, Collinson M, Hill RE (1997). Two enhancer domains control early aspects of the complex expression pattern of Msx1. Mech Dev.

[pbio-0040313-b057] Hartley KO, Hardcastle Z, Friday RV, Amaya E, Papalopulu N (2001). Transgenic *Xenopus* embryos reveal that anterior neural development requires continued suppression of BMP signaling after gastrulation. Dev Biol.

[pbio-0040313-b058] Golden JA, Bracilovic A, McFadden KA, Beesley JS, Rubenstein JL (1999). Ectopic bone morphogenetic proteins 5 and 4 in the chicken forebrain lead to cyclopia and holoprosencephaly. Proc Natl Acad Sci U S A.

[pbio-0040313-b059] Furuta Y, Piston DW, Hogan BL (1997). Bone morphogenetic proteins (BMPs) as regulators of dorsal forebrain development. Development.

[pbio-0040313-b060] Pierani A, Brenner-Morton S, Chiang C, Jessell TM (1999). A sonic hedgehog-independent, retinoid-activated pathway of neurogenesis in the ventral spinal cord. Cell.

[pbio-0040313-b061] Briscoe J, Sussel L, Serup P, Hartigan-O'Connor D, Jessell TM (1999). Homeobox gene *Nkx2.2* and specification of neuronal identity by graded Sonic hedgehog signalling. Nature.

[pbio-0040313-b062] Ericson J, Rashbass P, Schedl A, Brenner-Morton S, Kawakami A (1997). Pax6 controls progenitor cell identity and neuronal fate in response to graded Shh signaling. Cell.

[pbio-0040313-b063] Sasai Y, Lu B, Steinbeisser H, Geissert D, Gont LK (1994). *Xenopus* chordin: A novel dorsalizing factor activated by organizer-specific homeobox genes. Cell.

[pbio-0040313-b064] Janssen R, Prpic NM, Damen WG (2004). Gene expression suggests decoupled dorsal and ventral segmentation in the millipede Glomeris marginata (Myriapoda: Diplopoda). Dev Biol.

[pbio-0040313-b065] Bai CB, Stephen D, Joyner AL (2004). All mouse ventral spinal cord patterning by hedgehog is Gli dependent and involves an activator function of Gli3. Dev Cell.

[pbio-0040313-b066] Lei Q, Zelman AK, Kuang E, Li S, Matise MP (2004). Transduction of graded Hedgehog signaling by a combination of Gli2 and Gli3 activator functions in the developing spinal cord. Development.

[pbio-0040313-b067] Litingtung Y, Chiang C (2000). Specification of ventral neuron types is mediated by an antagonistic interaction between Shh and Gli3. Nat Neurosci.

[pbio-0040313-b068] Persson M, Stamataki D, te Welscher P, Andersson E, Bose J (2002). Dorsal-ventral patterning of the spinal cord requires Gli3 transcriptional repressor activity. Genes Dev.

[pbio-0040313-b069] Wijgerde M, McMahon JA, Rule M, McMahon AP (2002). A direct requirement for Hedgehog signaling for normal specification of all ventral progenitor domains in the presumptive mammalian spinal cord. Genes Dev.

[pbio-0040313-b070] Conway-Morris S (1998). Early metazoan evolution: Reconciling paleontology and molecular biology. Amer Zool.

[pbio-0040313-b071] Conway-Morris S (2003). The Cambrian “explosion” of metazoans and molecular biology: Would Darwin be satisfied?. Int J Dev Biol.

[pbio-0040313-b072] Lindsley DL, Zimm GG (1992). The Genome of *Drosophila melanogaster*.

[pbio-0040313-b073] Yamada T, Placzek M, Tanaka H, Dodd J, Jessell TM (1991). Control of cell pattern in the developing nervous system: Polarizing activity of the floor plate and notochord. Cell.

[pbio-0040313-b074] Robertson CP, Gibbs SM, Roelink H (2001). cGMP enhances the sonic hedgehog response in neural plate cells. Dev Biol.

[pbio-0040313-b075] Ashe HL, Levine M (1999). Local inhibition and long-range enhancement of Dpp signal transduction by Sog. Nature.

